# Ultrasonographic Halo Score in giant cell arteritis: association with intimal hyperplasia and ischaemic sight loss

**DOI:** 10.1093/rheumatology/keaa806

**Published:** 2020-12-23

**Authors:** Kornelis S. M. van der Geest, Konrad Wolfe, Frances Borg, Alwin Sebastian, Abdul Kayani, Alessandro Tomelleri, Prisca Gondo, Wolfgang A. Schmidt, Raashid Luqmani, Bhaskar Dasgupta

**Affiliations:** 1 Department of Rheumatology and Clinical Immunology, University of Groningen, University Medical Center Groningen, Groningen, The Netherlands; 2 Department of Rheumatology; 3 Department of Pathology, Southend University Hospital, Westcliff-on-sea,UK; 4 Medical Centre for Rheumatology Berlin-Buch, Immanuel Krankenhaus Berlin, Berlin, Germany; 5 Department of Rheumatology, NDORMS, Nuffield Orthopaedic Centre, University of Oxford, Oxford, UK

**Keywords:** vasculitis, giant cell arteritis, imaging, ultrasound, biopsy

## Abstract

**Objectives:**

We investigated the relationship between the ultrasonographic Halo Score and temporal artery biopsy (TAB) findings in GCA.

**Methods:**

This is a prospective study including 90 patients suspected of having GCA. Ultrasonography of temporal/axillary arteries and a TAB were obtained in all patients at baseline. An experienced pathologist evaluated whether TAB findings were consistent with GCA, and whether transmural inflammation, giant cells and intimal hyperplasia were present. Ultrasonographic Halo Scores were determined. Receiver operating characteristic analysis was performed.

**Results:**

Twenty-seven patients had a positive TAB, while 32 patients with a negative TAB received a clinical diagnosis of GCA after 6 months of follow-up. Patients with a positive TAB showed higher Halo Scores than patients with a negative TAB. The presence of intimal hyperplasia in the biopsy, rather than the presence of transmural inflammation or giant cells, was associated with elevated Halo Scores in patients with GCA. The Halo Score discriminated well between TAB-positive patients with and without intimal hyperplasia, as indicated by an area under the curve of 0.82 in the receiver operating characteristic analysis. Patients with a positive TAB and intimal hyperplasia more frequently presented with ocular ischaemia (40%) than the other patients with GCA (13–14%).

**Conclusion:**

The ultrasonographic Halo Score may help to identify a subset of GCA patients with intimal hyperplasia, a TAB feature associated with ischaemic sight loss.

**Rheumatology key messages**

- The ultrasonographic Halo Score identifies a subset GCA patients with intimal hyperplasia.
- Ultrasonography might be less effective in detecting arterial inflammation in the absence of intimal hyperplasia.
- Intimal hyperplasia is a biopsy feature associated with ischaemic sight loss in GCA.

## Introduction

GCA is the most common form of primary vasculitis in the elderly. Inflammation of cranial arteries may compromise the blood flow thereby causing ischaemic complications [1]. Prior studies have linked temporal artery biopsy (TAB) findings, such as the presence of intimal hyperplasia, to a high risk of cranial ischaemic symptoms [2, 3]. It has been proposed that distinct disease subsets may exist within GCA [4].

Ultrasonography of the temporal and axillary arteries is recommended as the first-line investigation in the diagnostic work-up of GCA, and it may reveal the presence of one or more halos [5, 6]. A halo is a hypo-echoic thickening of the arterial wall, and in particular of the intima-media complex [7]. We recently developed an ultrasonographic Halo Score, which quantifies the extent of arterial inflammation in patients with GCA [8]. We noted that patients with high Halo Scores more frequently presented with ischaemic sight loss at diagnosis than patients with low Halo Scores [8].

We hypothesized that the ultrasonographic Halo Score might identify a subset of GCA patients with intimal hyperplasia, a TAB feature associated with ischaemic sight loss. We therefore investigated the relationship between the ultrasonographic Halo Score, biopsy findings and the occurrence of ocular ischaemia.

## Methods

### Patients

Patients suspected of having GCA (*n* = 92) were prospectively recruited at the Rheumatology Department of Southend University Hospital between June 2010 and December 2013 as part of the Temporal Artery Biopsy vs ULtrasound in diagnosis of giant cell arteritis (TABUL) study [9]. All patients underwent arterial ultrasonography followed by a TAB. The final clinical diagnosis was established after 6 months in all except for three patients. Two patients without follow-up and with a negative TAB were excluded due to uncertainty about the diagnosis. One patient without follow-up but with a positive TAB was retained in the analysis. The study was performed in accordance with the declaration of Helsinki. All patients provided written informed consent. The study was approved by the Berkshire Research Ethics Committee (REC#09/H0505/132).

### Ultrasound

As previously described [8], ultrasonography of temporal arteries (i.e. common superficial, parietal and frontal segments) and axillary arteries was performed within 7 days of initiation of high-dose glucocorticoids by a single experienced sonographer (B.D.) with an Esaote MyLab70 or MyLabTwice. A linear probe (LA435) with a grey-scale frequency of 18 MHz and colour Doppler frequency of 9 MHz was used. A halo sign was morphologically defined as an US finding of a dark hypoechoic area around the vessel lumen. Halo Scores were calculated as shown in supplementary Fig. S1, available at *Rheumatology* online. In brief, the maximum thickness of each halo was recorded, and graded according to previously reported grading system [8]. The sum of halo grades in all temporal artery segments and axillary arteries was used to calculate the Halo Score. Separate Halo Scores were calculated for the temporal artery segments only (i.e. temporal artery Halo Score) and the axillary arteries only (i.e. axillary artery Halo Score).

### Temporal artery biopsy

TAB was performed within 14 days of initiation of high-dose glucocorticoids. The TAB was evaluated by an experienced pathologist for the presence of a transmural infiltrate, giant cells and intimal hyperplasia. The pathologist also reported whether or not the biopsy findings were consistent with GCA. The pathologist was unaware of the clinical and US findings.

### Statistics

The Mann–Whitney *U* test was used for comparison of continuous variables in two independent groups. If more groups were compared, the Mann–Whitney *U* test was preceded by the Kruskal–Wallis test. χ^2^ test for trend was used to evaluate the occurrence of ocular ischaemia among different groups of patients stratified according to TAB findings. Receiver operating characteristic analysis with area under the curve was performed. Optimal cut-off points were determined according to the Youden Index. The sensitivity, specificity, positive likelihood ratio and negative likelihood ratio at the optimal cut-off point were determined. Multiple linear regression was performed with backward exclusion of predicting variables. The probability of F for removal was 0.10. Halo Scores were used as the dependent variable. Predicting variables were: sex: 0 = female, 1 = male; transmural inflammation, giant cells, intimal hyperplasia: 0 = absent, 1 = present. R squared (R^2^) was reported. Due to non-normal distribution, Halo Scores were transformed by square root. Normality of residuals was tested by histograms and P-P plots. Linearity and homoscedasticity was tested by scatter plots and P-P plots. Multicollinearity was excluded by Pearson correlation coefficient <0.7, variance inflation factor <10, tolerance statistics >0.2 and Collinearity Diagnostics. Data were analysed with IBM SPSS Statistics 25, StatsDirect 3.1.22 and Graphpad Prism 5. *P*-values <0.05 were considered statistically significant.

## Results

### Patients’ characteristics and TAB results

Overall, 90 patients were included in the study analysis. The TAB was positive for GCA in 27 patients (37% males, median age 78 years) as shown in supplementary Table S1, available at *Rheumatology* online. Thirty-two patients with a negative TAB at baseline received a final clinical diagnosis of GCA after 6 months follow-up (16% males, median age 73). The remaining patients were classified as non-GCA patients (36% males, median age 67 years). Ocular ischaemia was evaluated and defined by the presence of anterior ischaemic optic neuropathy, posterior ischaemic optic neuropathy and/or a relative afferent pupillary defect. Ocular ischaemia was present in 13% of patients with TAB-negative GCA and in 33% of patients with TAB-positive GCA. Non-arteritic ocular ischaemia was observed in 23% of non-GCA patients. Transmural inflammation and giant cells were observed in 18 and 24 positive TABs, respectively. Overall, 26 out of 27 positive TABs contained a transmural infiltrate and/or giant cells. The other positive TAB contained an adventitial infiltrate, disruption of the internal elastic lamina and intimal hyperplasia. Intimal hyperplasia was present in 20 patients with a positive TAB, and in 2 patients with a negative TAB. The latter two patients showed Halo Scores of 0 and received a final clinical diagnosis of GCA after 6 months follow-up (supplementary Table S2, available at *Rheumatology* online).

### Relationship between halo score and TAB findings

**Fig. 1:**
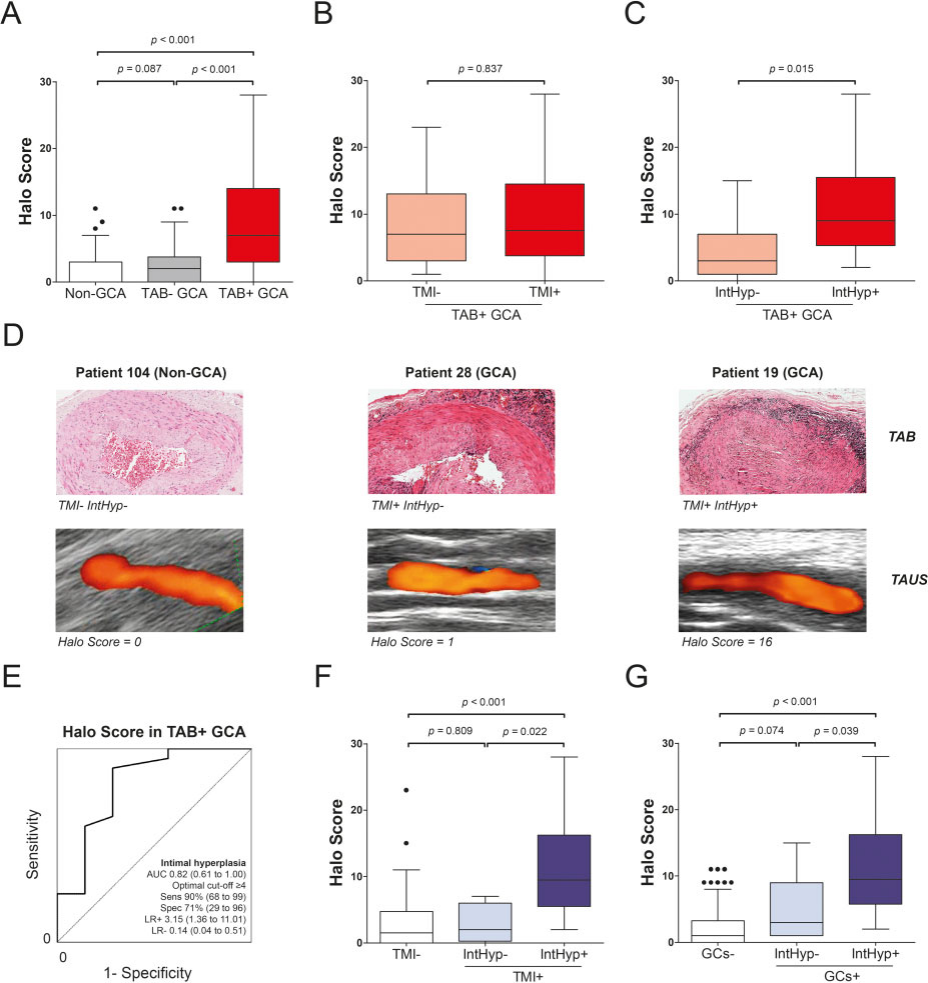
Ultrasonographic Halo Score and TAB findings at diagnosis (**A**) Halo Score in non-GCA patients (*n* = 31), patients with TAB-negative GCA (*n* = 32) and TAB-positive GCA (*n* = 27). (**B**) Halo Score in patients with TAB-positive GCA with TMI (*n* = 18) and without transmural inflammation (*n* = 9). (**C**) Halo Score in patients with TAB-positive GCA with IntHyp (*n* = 20) and without intimal hyperplasia (*n* = 7). (**D**) Paired TAB and TAUS images of three patients. (**E**) ROC curve showing the ability of Halo Score to discriminate between patients with and without intimal hyperplasia among those with TAB positive GCA (*n* = 27). The optimal cut-off point was determined by the Youden Index. Diagnostic accuracy parameters are shown with their 95% CIs. (**F**) Halo Scores in patients (*n* = 90) stratified according to the presence of IntHyp and TMI (TMI−, *n* = 72; TMI+ IntHyp−, *n* = 4; TMI+ IntHyp+, *n* = 14), or (**G**) GCs (GCs−, *n* = 66; GCs+ IntHyp−, *n* = 6; GCs+ IntHyp+, *n* = 18). Statistical significance by Mann–Whitney *U* test is shown. If more than three groups were compared, the latter test was preceded by the Kruskal–Wallis test. AUC: area under the curve; GCs: giant cells; IntHyp: intimal hyperplasia; LR+: positive likelihood ratio; LR−: negative likelihood ratio; ROC: receiver operating characteristic; Sens: sensitivity; Spec: specificity; TAB: temporal artery biopsy; TAUS: temporal artery ultrasound; TMI: transmural inflammation.

Patients with TAB-negative GCA tended to have higher Halo Scores at baseline than non-GCA patients, albeit not statistically significant; whereas patients with TAB-positive GCA showed markedly higher Halo Scores than both these groups (Fig. 1A). The latter finding could be explained by elevated temporal artery Halo Scores, and to some extent axillary artery Halo Scores, in patients with TAB positive GCA (supplementary Fig. S2A and B, available at *Rheumatology* online). We next investigated the relationship between the ultrasonographic Halo Score and specific biopsy features in patients with TAB-positive GCA. The presence of transmural inflammation in the TAB showed no association with Halo Scores in these patients (Fig. 1B). The impact of giant cells could not be genuinely evaluated, since these cells were present in nearly all positive TABs. However, the presence of intimal hyperplasia was strongly associated with elevated Halo Scores in patients with TAB-positive GCA (Fig. 1C and D). Temporal artery Halo Scores, rather than axillary artery Halo Scores, tended to be higher in patients with TAB-positive GCA and intimal hyperplasia (supplementary Fig. S2C and D, available at *Rheumatology* online). Overall, Halo Scores could discriminate well between TAB-positive GCA patients with and without intimal hyperplasia (Fig. 1E). Multiple linear regression analysis confirmed that the presence of intimal hyperplasia, but not of transmural inflammation or giant cells, was an independent predictor for higher Halo Scores (B = 6.548; 95% CI 2.140 to 12.742; *P* = 0.001) in patients with TAB-positive GCA (supplementary Table S3, available at *Rheumatology* online). When all 90 patients were evaluated, the presence of intimal hyperplasia remained the key TAB feature associated with elevated Halo Scores (Fig. 1F and G).

### Intimal hyperplasia and ischaemic sight loss

**Fig. 2:**
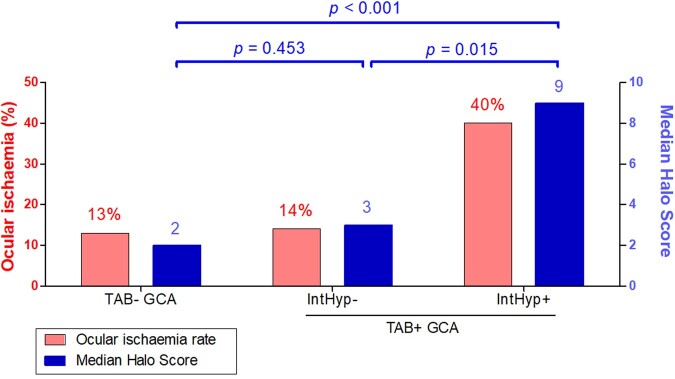
Halo Score, TAB findings and ocular ischaemia The percentage of patients with ocular ischaemia and median Halo Scores are shown for patients with TAB-negative GCA (*n* = 32), patients with TAB-positive GCA without intimal hyperplasia (*n* = 7) and patients with TAB-positive GCA and intimal hyperplasia (*n* = 20). The relationship between biopsy findings and the percentage of patients with ocular ischaemia was statistically significant, as indicated by the χ^2^ for trend = 5.185, d.f. = 1, *P* = 0.023. Statistical significance by Mann–Whitney *U* test of Halo Scores is shown. Since more than three groups were compared, the latter test was preceded by the Kruskal–Wallis test. IntHyp: intimal hyperplasia; TAB: temporal artery biopsy.

We next investigated the relationship between intimal hyperplasia and the occurrence of ocular ischaemia in patients with GCA (Fig. 2). The occurrence of ischaemic sight loss was lowest in patients with a negative TAB (4 out 32 patients; 13%), slightly higher in those with a positive TAB lacking intimal hyperplasia (1 out of 7 patients; 14%) and substantially higher in patients with a positive TAB and intimal hyperplasia (8 out of 20 patients; 40%). The percentage of patients with ocular ischaemia showed a statistically significant relationship with the respective TAB abnormalities (χ^2^ for trend = 5.185, d.f.  = 1, *P* = 0.023). Halo Scores showed an increase proportionate to the occurrence of ocular ischaemia across the three GCA groups.

## Discussion

This is the first study linking a specific biopsy feature, i.e. intimal hyperplasia, to elevated ultrasonographic Halo Scores in patients with GCA. We confirm that intimal hyperplasia associates with GCA-related sight loss. Thus, Halo Scores may identify a subset of GCA patients with intimal hyperplasia and high rates of ischaemic sight loss.

Patients with a positive TAB and intimal hyperplasia showed the most extensive arterial wall swelling on US, as indicated by high Halo Scores. This finding supports the notion that ultrasonographic halos primarily reflect thickening of the intima-media complex, and in particular of the intima [10, 11]. An earlier study indicated that halos are linked to the presence of transmural infiltrates in the TAB, but the impact of intimal hyperplasia was not evaluated [12]. Our findings suggest that Halo Scores are strongly associated with intimal hyperplasia in patients with GCA. Transmural inflammation in the absence of intimal hyperplasia was not associated with higher Halo Scores, and might thus be difficult to diagnose by ultrasonography.

Patients with a positive TAB and intimal hyperplasia showed the highest prevalence of ischaemic sight loss at presentation (40%). This observation is in agreement with two prior reports, which linked intimal hyperplasia to a high rate of neuro-ophthalmic, ischaemic complications [2, 3]. Intimal hyperplasia is caused by proliferation of myofibroblasts in the intimal layer of the arterial wall. These cells are derived from activated vascular smooth muscle cells in the medial layer [13]. High-grade intimal hyperplasia may directly compromise the luminal blood flow, thereby causing ischaemic complications in the downstream tissues [1]. The presence of intimal hyperplasia in the temporal artery is likely paralleled by intimal hyperplasia in other arterial regions affected by GCA, which reflects the systemic nature of this disease. This would explain why intimal hyperplasia in the TAB associates with the ultrasonographic Halo Score and the occurrence of ocular ischaemia.

Strengths of our study include its prospective design with respect to performance of both ultrasound and TAB. Our study may also have limitations. The interrater reliability was not assessed, but ultrasonography was done by an experienced sonographer and the quality of the US images and reports was monitored by an expert panel [9]. Excellent interrater reliability for intimal hyperplasia has been reported by pathologists from our centre [3]. The median TAB length was <1 cm and patchy changes might have been missed. The TAB was performed after initiation of high-dose glucocorticoid treatment in many patients, as it was considered unethical to withhold treatment. However, the TAB remains diagnostic if performed within 2 weeks after initiation of treatment [14], and intimal hyperplasia typically persists beyond 2 weeks of treatment [15]. The number of patients was limited in several sub-groups that were analysed. Confirmation by larger studies is therefore needed.

In conclusion, the ultrasonographic Halo Score is strongly linked to the presence of intimal hyperplasia, a TAB finding associated with ocular ischaemia in GCA. Further studies are needed to evaluate the ability of ultrasonography to detect arterial inflammation in the absence of intimal hyperplasia.

## Supplementary Material

keaa806_Supplementary_Data
