# Supplementary material for: Ultrasonographic Halo Score in giant cell arteritis: association with intimal hyperplasia and ischaemic sight loss
Source: Rheumatology (Oxford). 2020 Dec 23;60(9):4361–6. doi: 10.1093/rheumatology/keaa806 (PMC8410002; doi:10.1093/rheumatology/keaa806)
Supplement: keaa806_Supplementary_Data [file keaa806_supplementary_data.docx]

# Supplementary Tables

**Supplementary Table S1. Patients’ characteristics.** Data are provided for 90 patients suspected of having GCA. Ocular ischemia was defined by the presence of anterior ischemic optic neuropathy, posterior ischemic optic neuropathy and/or relative afferent pupillary defect. ^§^ Pre-treatment CRP was determined in 55 patients, including 13 non-GCA patients, 18 patients with TAB negative GCA, and 24 patients with TAB positive GCA. TAB = temporal artery biopsy.

| **Patients’ characteristics** | **Non-GCA** | **TAB negative GCA** | **TAB positive GCA** |
| --- | --- | --- | --- |
| Total no. | 31 | 32 | 27 |
| Sex, no. of males | 11 (36%) | 5 (16%) | 10 (37%) |
| Age, median (range) years | 67 (44-90) | 73 (50-96) | 78 (63-92) |
| Fulfilling 1990 ACR criteria for GCA, no of patients | 22 (71%) | 24 (75%) | 27 (100%) |
| Ocular ischemia present, no. of patients | 7 (23%) | 4 (13%) | 9 (33%) |
| CRP, mg/L, median (range) | 13 (3-205) ^§^ | 40 (12-174) ^§^ | 77 (3-329) ^§^ |
| Haemoglobin, (g/dL), median (range) | 13.5 (10.1-16.0) | 12.6 (10.3-15.5) | 11.7 (8.9-14.7) |
| Platelets, 10^9^/L, median (range) | 317 (126-522) | 333 (167-636) | 451 (224-661) |
| High dose GCs prior to US, no. of days, median (range) | 2 (0-6) | 2 (0-7) | 1 (0-6) |
| High dose GCs prior to TAB, no. of days, median (range) | 6 (2-9) | 7 (2-14) | 5 (0-11) |
| TAB length, median (range) mm | 8 (2-13) | 5 (3-20) | 8 (2-20) |
| TAB transmural infiltrate present, no. of patients | 0 (0%) | 0 (0%) | 18 (67%) |
| TAB giant cells present, no. of patients | 0 (0%) | 0 (0%) | 24 (89%) |
| TAB intimal hyperplasia present, no. of patients | 0 (0%) | 2 (6%) | 20 (74%) |

**Supplementary Table S2. Characteristics of two patients with a negative TAB containing intimal hyperplasia.** TAB = temporal artery biopsy.

| **Characteristics** | **Patient EP-12-005** | **Patient EP-12-050** |
| --- | --- | --- |
| Sex | Female | Female |
| Age (years) | 96 | 72 |
| Clinical diagnosis after 6 months follow-up | GCA | GCA |
| Ocular ischaemia | Absent | Absent |
| TAB result | Negative | Negative |
| Transmural infiltrate | Absent | Absent |
| Giant cells | Absent | Absent |
| Intimal hyperplasia | Present | Present |
| Halo Score | 0 | 0 |

**Supplementary Table S3. Variables predicting Halo Scores in patients with TAB positive GCA.** Data are shown for baseline Halo Scores in patients with positive TAB (n=27). Multiple linear regression analysis was performed with backward exclusion of predicting variables. Since the Halo Score was not normally distributed, the Halo Score was transformed by square root. The probability of F for removal was 0.10. Results of the final model are shown. Sex: 0 = female, 1= male. Transmural inflammation, giant cells and intimal hyperplasia on TAB: 0 = absent, 1 = present. (-) Variable removed due to backward exclusion. ^a^ R^2^ = 0.391, F (2,24) = 7.705, *p* = 0.003.

| **Dependent variable** | **Predicting variable** | **Final model of multiple linear regression**  **B (95% CI)** | ***p* value** |
| --- | --- | --- | --- |
| Halo Score | Male sex | 3.232 (0.308 to 7.626) ^a^ | 0.026 |
|  | Transmural inflammation | - |  |
|  | Giant cells | - |  |
|  | Intimal hyperplasia | 6.548 (2.140 to 12.742) ^a^ | 0.001 |

# Supplementary Figures

**Supplementary Figure S1. Ultrasonographic Halo Score.** The three segments of the temporal artery (TA) and the axillary artery (AA) are bilaterally examined by ultrasonography [1]. If present, the halo thickness is measured with one decimal place at the point of maximum thickness in the longitudinal plane. Halo thickness in each arterial segments is graded. The sum of all halo grades provides the Halo Score. Halo grades in the axillary artery are multiplied by a factor of 3 in order to give equal weight to temporal and axillary arteries. Separate TA and AA Halo Scores can be calculated as well. *Figure was created with Biorender.*


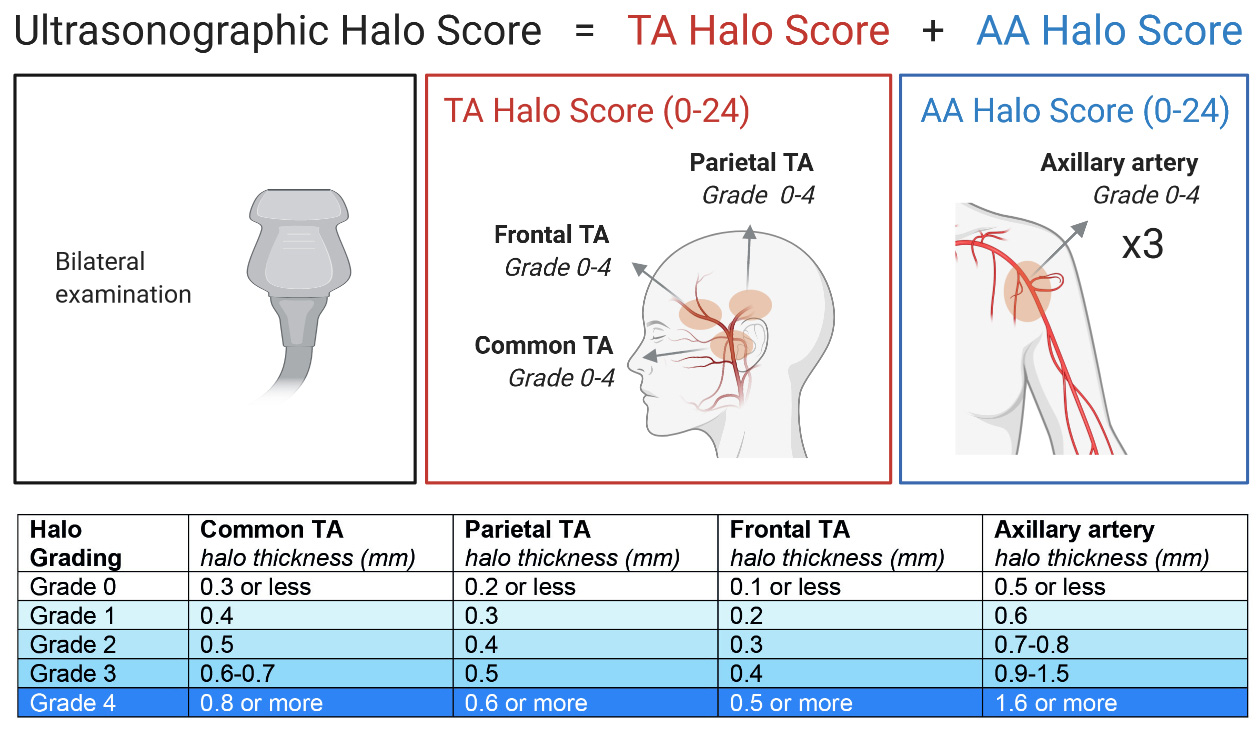


**Supplementary Figure S2. Temporal and axillary artery Halo Scores.** (A) Temporal artery (TA) and (B) axillary artery (AA) Halo Scores in non-GCA patients, patients with TAB negative GCA and TAB positive GCA. (C) TA Halo Score and (D) AA Halo Scores in patients with TAB positive GCA with intimal hyperplasia and without intimal hyperplasia. Data are shown for same patients as shown in Figure 1A and 1B. Statistical significance by Mann Whitney U test is shown. If more than three groups were compared, the latter test was preceded by the Kruskal-Wallis test. IntHyp = intimal hyperplasia. TAB = temporal artery biopsy.


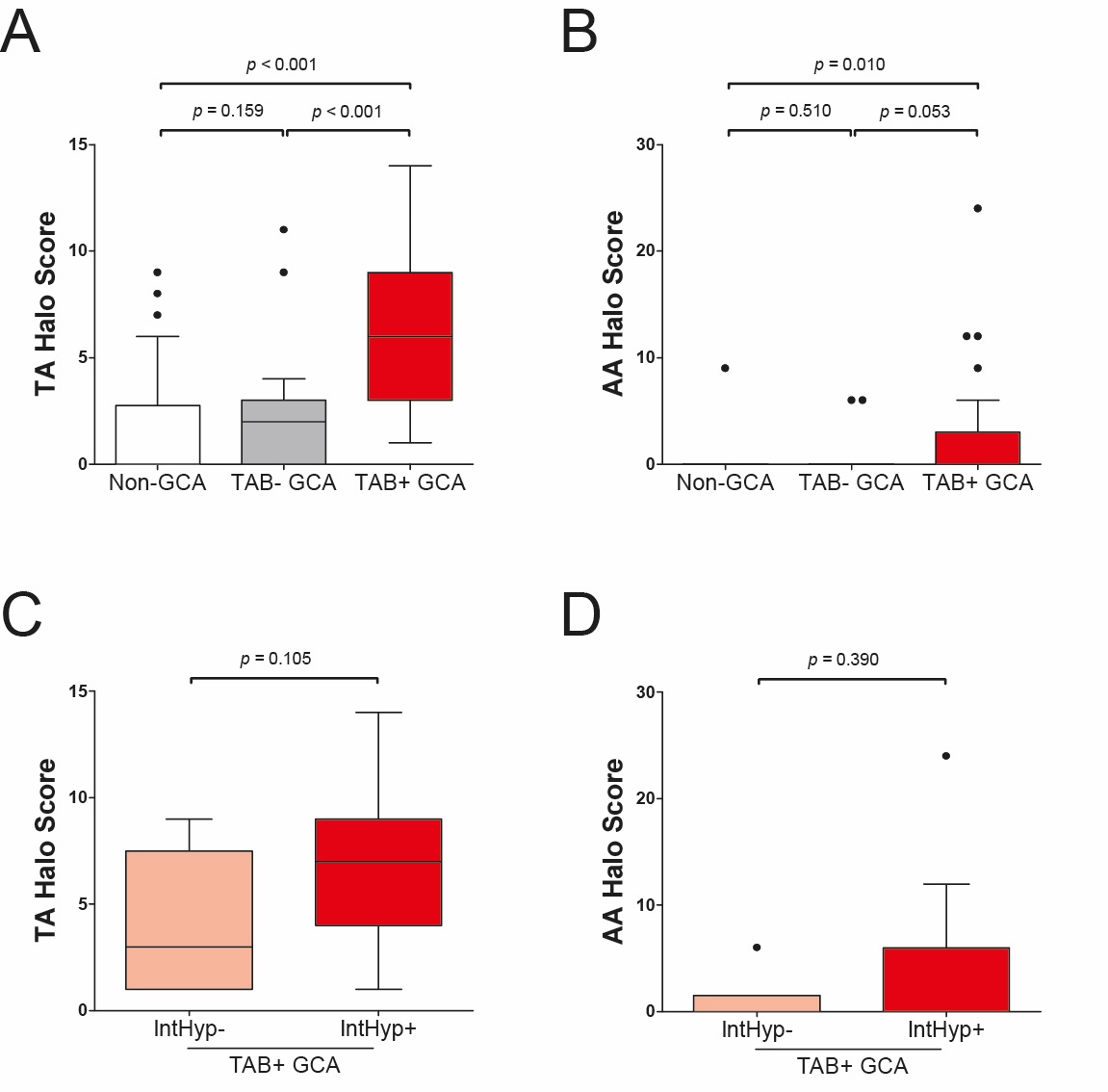


**References**

1. van der Geest KSM, Borg F, Kayani A, Paap D, Gondo P, Schmidt W, et al. Novel ultrasonographic Halo Score for giant cell arteritis: assessment of diagnostic accuracy and association with ocular ischaemia. Annals of the Rheumatic Diseases 2020 Jan 3;79(3):393-399.
